# Editorial: The model of Ramadan diurnal intermittent fasting: unraveling the health implications, volume III

**DOI:** 10.3389/fnut.2025.1586573

**Published:** 2025-04-11

**Authors:** MoezAlIslam Ezzat Faris, Meghit Boumediene Khaled, Hamdi Chtourou, Faiza Kalam, Dana N. Abdelrahim, Ahmed S. BaHammam

**Affiliations:** ^1^Department of Clinical Nutrition and Dietetics, Applied Science Private University, Amman, Jordan; ^2^Faculté des Sciences de la Nature et de la Vie, Univeristé Djillali Liabes Sidi Bel Abbès, Sidi Bel Abbes, Algeria; ^3^High Institute of Sport and Physical Education of Sfax, Sfax, Tunisia; ^4^Division of Cancer Prevention and Control, Department of Internal Medicine, The Ohio State University, Columbus, OH, United States; ^5^The Ohio State University Comprehensive Cancer Center, The Ohio State University, Columbus, OH, United States; ^6^University of Sharjah, Sharjah, United Arab Emirates; ^7^University Sleep Disorders Center, King Saud University, Riyadh, Saudi Arabia

**Keywords:** fasting, intermittent fasting, caloric restriction, Ramadan, Ramadan fasting

## Introduction

Intermittent fasting (IF) has emerged as a widely studied dietary practice with substantial metabolic and physiological benefits, including improved insulin sensitivity, weight regulation, reduced inflammation, and enhanced circadian rhythm synchronization (1). Among various intermittent fasting (IF) regimens, Ramadan intermittent fasting (RIF) presents a unique model due to its structured, religiously mandated practice, which involves daily fasting from dawn to sunset for a month. Observed by nearly 1.5 billion Muslims globally, RIF provides an invaluable natural framework for studying the implications of diurnal fasting on human health (2). Despite extensive research on fasting, RIF offers distinct insights due to its periodic dry fasting nature, which differentiates it from water-based intermittent fasting protocols (3, 4).

This Research Topic, “*The model of Ramadan diurnal intermittent fasting: unraveling the health implications – volume III*,” presents a collection of cutting-edge studies that explore the physiological, metabolic, behavioral, and clinical effects of RIF. By integrating findings from various populations and health conditions, this body of research contributes to the growing literature on fasting. It provides evidence to optimize fasting-based interventions for diverse health outcomes. The articles featured in this Research Topic collectively highlight the role of RIF in shaping metabolic health, disease prevention, and overall wellbeing.

## The impact of Ramadan fasting on health

The growing interest in RIF has catalyzed research into its multifaceted impact on human physiology. One of the most significant findings is the role of RIF in enhancing metabolic health, characterized by favorable changes in glucose homeostasis, lipid metabolism, and body composition (5). Studies have demonstrated that fasting-induced metabolic shifts can be leveraged to manage conditions such as diabetes, obesity, and cardiovascular disease; however, individual variability necessitates further exploration (6–8).

Beyond metabolic regulation, RIF has shown promising effects on inflammatory and oxidative stress markers, reinforcing its potential as a preventive strategy against chronic diseases (9). Given that oxidative stress plays a critical role in aging and various pathologies, the observed improvements in antioxidant pathways during RIF underscore its broader health benefits. Moreover, emerging research on gene expression suggests that RIF modulates pathways associated with autophagy, circadian rhythm regulation, longevity, and stress response mechanisms, offering novel insights into its systemic effects (10–12).

## Key themes in this Research Topic

This Research Topic encompasses a diverse range of studies examining various aspects of RIF, thereby further solidifying its importance in the health sciences. The included articles collectively enhance our understanding of the effects of fasting on various health parameters without being confined to metabolic benefits alone.

The 13 studies in this Research Topic provide comprehensive insights into the diverse health implications of RIF. Conducted by 82 authors from 15 countries, these studies examine the RIF's effects on metabolic health (validating risk assessment tools for diabetic patients (Alketbi et al.), assessing benefits for NAFLD (Lin et al.), metabolic profile, and blood pressure (Al-Jafar et al.), hormonal adaptations (in pre-and post-menopausal women) (Al Zunaidy et al.) and effect of calorie restriction and intermittent fasting, including RIF, on PCOS (Kalsekar et al.). Effect on physical performance [timing of resistance training (Triki et al.), impact on bioenergetic (Özbay et al.) pathways during exercise], molecular mechanisms (gene expression related to inflammation and oxidative stress, influence of haptoglobin polymorphism) (Madkour et al.), nutritional interventions (benefits of Ramadan-specific nutrition education) (Gul et al.), physiological adaptations (changes in body water compartments) (Najafi et al.), gut microbiome (Saglam et al.) alterations (shifts in bacterial composition and correlations with dietary components), as well as the changes in inflammatory markers and mental health (Ghashang et al.) parameters. Furthermore, the effect of combining exercise and fasting on animal models of osteoporosis (Albrahim et al.) was examined. Together, these studies highlight the potential of RIF to improve metabolic health, including inflammatory markers and body composition, while emphasizing that these benefits result from a combination of fasting and dietary and lifestyle habits during non-fasting hours.

One critical area explored is the role of RIF in managing chronic diseases. Research examining the application of RIF in diabetes risk assessment highlights the need for personalized medical guidance for individuals with type 2 diabetes who fast. Similarly, findings on cardiovascular risk markers highlight the potential of RIF in modulating lipid profiles and blood pressure, reinforcing its relevance in cardiometabolic health strategies (Madkour et al.).

Another emerging theme is the interaction between fasting and physical performance. Studies investigating resistance training and exercise timing during Ramadan provide valuable insights into optimizing muscle function and hormonal balance in athletes and active individuals. The debate over training in the fasting vs. fed state remains ongoing, and contributions to this Research Topic offer evidence-based recommendations for maintaining strength and performance during fasting periods [(13, 14); Triki et al.].

Furthermore, this volume expands on the relationship between fasting and the gut microbiome, an area of increasing scientific interest. The gut microbiota plays a pivotal role in maintaining metabolic health, regulating immune function, and controlling inflammation. This Research Topic suggests that RIF induces shifts in microbial composition, with potential implications for long-term gut health (Saglam et al.). These findings highlight the need for further investigations into dietary modifications during RIF to optimize gut microbiome adaptations and overall health benefits.

## Future directions in Ramadan and health research

While the studies in this Research Topic offer significant advancements in understanding RIF, several key areas require further investigation. Future research should prioritize:

1. **Longitudinal studies on RIF**: most existing research focuses on the short-term effects of fasting. Long-term follow-up studies are needed to evaluate whether RIF-induced metabolic adaptations persist beyond Ramadan and contribute to long-term health benefits or risks.
2. **Personalized approaches to fasting**: given individual differences in metabolic responses, genetics, age, and health status, future research should explore personalized fasting protocols that maximize benefits while minimizing potential adverse effects.
3. **Molecular and systems biology investigations**: advanced omics technologies, including metabolomics, lipidomics, proteomics, and epigenetics, should be leveraged to elucidate the precise mechanisms through which RIF influences cellular function and disease prevention.
4. **Comparative studies with other IF models**: although RIF shares similarities with other IF protocols, it differs in duration, hydration status, and cultural context. Comparative studies can help delineate its unique effects and guide recommendations for those interested in adopting IF beyond Ramadan.
5. **Clinical applications and public health policies**: the integration of RIF into clinical and public health recommendations requires robust evidence. Studies on fasting interventions in patient populations, particularly those with chronic illnesses, are crucial for informing guidelines and policy decisions.

## Conclusion

Ramadan intermittent fasting serves as a valuable model for understanding the broader implications of intermittent fasting on human health. This Research Topic presents compelling evidence that RIF extends beyond religious observance and represents a structured dietary intervention with diverse physiological benefits. The insights gained from these studies pave the way for refining fasting strategies to optimize health outcomes in various populations. As interest in IF continues to grow, future research should strive to bridge existing knowledge gaps, ensuring that fasting recommendations are evidence-based, personalized, and accessible to those seeking to harness its health-promoting potential.

## References

[1] Obaideen K Shihab KHA Madkour MI Faris ME. Seven decades of Ramadan intermittent fasting research: bibliometrics analysis, global trends, and future directions. Diabetes Metab Syndr. (2022) 16:102566. 10.1016/j.dsx.2022.102566 35872466

[2] Madkour M Giddey AD Soares NC Semreen MH Bustanji Y Zeb F. Ramadan diurnal intermittent fasting is associated with significant plasma metabolomics changes in subjects with overweight and obesity: a prospective cohort study. Front Nutr. (2023) 9:1008730. 10.3389/fnut.2022.1008730 36698470 PMC9868699

[3] Musharrat F Akheruzzaman M Khanam J Amin MR. Effects of wet and dry intermittent fasting on weight and cardiovascular risk indicators. Biores Commun. (2021) 8:1053–1060. 10.3329/brc.v8i1.57044

[4] BaHammam AS Pirzada A. Timing matters: the interplay between early mealtime, circadian rhythms, gene expression, circadian hormones, and metabolism—a narrative review. Clocks Sleep. (2023) 5:507–35. 10.3390/clockssleep5030034 37754352 PMC10528427

[5] Jahrami HA Faris ME Janahi A Janahi M Abdelrahim DN Madkour MI. Does four-week consecutive, dawn-to-sunset intermittent fasting during Ramadan affect cardiometabolic risk factors in healthy adults? A systematic review, meta-analysis, and meta-regression. Nutr, Metab Cardiov Dis. (2021) 31:2273–301. 10.1016/j.numecd.2021.05.002 34167865

[6] BaHammam AS Almeneessier AS. Recent evidence on the impact of ramadan diurnal intermittent fasting, mealtime, and circadian rhythm on cardiometabolic risk: a review. Front Nutr. (2020) 7:28. 10.3389/fnut.2020.00028 32219098 PMC7078334

[7] Faris MA Jahrami H BaHammam A Kalaji Z Madkour M Hassanein M. A systematic review, meta-analysis, and meta-regression of the impact of diurnal intermittent fasting during Ramadan on glucometabolic markers in healthy subjects. Diabetes Res Clin Pract. (2020) 165:108226. 10.1016/j.diabres.2020.108226 32446800

[8] Faris M Abdelrahim DN El Herrag SE Khaled MB Shihab KA AlKurd R. Cardiometabolic and obesity risk outcomes of dawn-to-dusk, dry intermittent fasting: Insights from an umbrella review. Clin Nutr ESPEN. (2025) 67:127–45. 10.1016/j.clnesp.2025.03.006 40081802

[9] Faris ME Jahrami HA Obaideen AA Madkour MI. Impact of diurnal intermittent fasting during Ramadan on inflammatory and oxidative stress markers in healthy people: Systematic review and meta-analysis. J Nutr Intermed Metab. (2019) 15:18–26. 10.1016/j.jnim.2018.11.005

[10] Madkour MI El-Serafi AT Jahrami HA Sherif NM Hassan RE Awadallah S. Ramadan diurnal intermittent fasting modulates SOD2, TFAM, Nrf2, and sirtuins (SIRT1, SIRT3) gene expressions in subjects with overweight and obesity. Diabetes Res Clin Pract. (2019) 155:107801. 10.1016/j.diabres.2019.107801 31356832

[11] Alasmari AA Alhussain MH Al-Khalifah AS Alshiban NM Alharthi R Alyami NM. Ramadan fasting model modulates biomarkers of longevity and metabolism in male obese and non-obese rats. Sci Rep. (2024) 14:28731. 10.1038/s41598-024-79557-y 39567585 PMC11579461

[12] Bou Malhab LJ Madkour MI Abdelrahim DN Eldohaji L Saber-Ayad MN Eid Abdel-Rahman WM. Dawn-to-dusk intermittent fasting is associated with overexpression of autophagy genes: a prospective study on overweight and obese cohort. Clin Nutr ESPEN. (2025) 65:209–17. 10.1016/j.clnesp.2024.11.002 39542136

[13] Drummond MDM Soares PSG Savoi LA Silva RAD. Fasting reduces satiety and increases hunger but does not affect the performance in resistance training. Biol Sport. (2023) 41:57–65. 10.5114/biolsport.2024.131814 38524818 PMC10955748

[14] Triki R Zouhal H Chtourou H Salhi I Jebabli N Saeidi A. Timing of resistance training during ramadan fasting and its effects on muscle strength and hypertrophy. Int J Sports Physiol Perform. (2023) 18:579–89. 10.1123/ijspp.2022-0268 37068775

